# High-intensity training vs. low-intensity training for patients with anxiety: a randomised controlled trial

**DOI:** 10.1186/s13063-026-09472-2

**Published:** 2026-01-27

**Authors:** Martin Bystad, Sveinung Rydland, Christian Bugge, Sissel Høgmo, Birgit Brøndbo, Renate Jacobsen, Lorena García-Fernández, Roberto Rodríguez-Jiménez, Verónica Romero-Ferreiro, Rolf Wynn

**Affiliations:** 1 Division of Substance Use and Mental Health, University Hospital of North Norway, Tromsø, Norway; 2 Department of Psychology, UiT The Arctic University of Norway, Tromsø, Norway; 3 CIBERSAM-ISCIII (Biomedical Research Networking Centre in Mental Health), Madrid, Spain; 4 Department of Clinical Medicine, Miguel Hernández University, San Juan, Alicante, Spain; 5 Department of Psychiatry, University Hospital of San Juan, San Juan, Alicante, Spain; 6 Faculty of Medicine, Complutense University of Madrid (UCM), Madrid, Spain; 7 Instituto de Investigación 12 de Octubre (imas12), Hospital Universitario, 12 de Octubre, Madrid, Spain; 8 Universidad Europea de Madrid, Madrid, Spain; 9 Department of Clinical Medicine, UiT The Arctic University of Norway, Tromsø, Norway

**Keywords:** Physical training, High-intensity, Low-intensity, Anxiety, RCT

## Abstract

**Background:**

This protocol was developed to describe the design of a randomised controlled trial that will examine the clinical efficacy of a 4-week comparison of high-intensity vs. low-intensity physical training for people suffering from anxiety. The hypothesis is that the high-intensity group will have greater benefit in terms of reduced anxiety symptoms, improved physical health (blood pressure) and better adherence.

**Methods:**

Thirty adults aged 18 to 70 years diagnosed with an anxiety disorder will be recruited for this study. Participants will be randomised into an intervention group (high-intensity training) and a control group (low-intensity training). Randomisation will be performed using counterbalanced block randomisation in a 1:1 ratio, stratified by sex. Both groups will perform 4 weeks of twice-weekly training supervised by an exercise physiologist. The primary outcome will be the total score on the Hospital Anxiety and Depression Scale (HADS) and the total score on the Beck Anxiety Inventory (BAI). The secondary outcomes include blood-pressure changes and adherence. Evaluations will be performed at baseline and following 4 weeks of the interventions, and 6 months after the termination of the intervention period (secondary endpoint).

**Discussion:**

By investigating the clinical efficacy of a 4-week training intervention, we hope to provide applicable and generalisable knowledge about the efficacy of physical training for people suffering from anxiety disorders.

**Trial registration:**

Clinical Trials NCT06881758. Registered on 17 March 2025.

**Supplementary Information:**

The online version contains supplementary material available at 10.1186/s13063-026-09472-2.

## Background

Anxiety disorders are very common and may severely impact the quality of life of those affected [1]. It has been estimated that approximately 15% of the Norwegian population fulfil the diagnostic criteria for one or more anxiety disorders [2]. Based on extensive population surveys, as many as 33.7% of individuals will experience an anxiety disorder at some point in their lifetime [3].

In recent years, the potential benefits of physical exercise for mental health have been highlighted [4]. Physical activity is considered important for physical and mental health and is associated with a number of health benefits, including reduced blood pressure, greater heart capacity, less pain, improved insulin response, deeper sleep, stronger bones and improved metabolic health [5]. The Norwegian health authorities recommend physical activity as a central initiative for improving public health [6]. Further, the World Health Organization as well as national guidelines in Norway recommend at least 75 minutes of high-intensity exercise or 150 minutes of moderate-intensity exercise per week or a combination of these two [7, 8].

However, it is important to emphasise the well-documented effects of physical activity on mental health disorders [5]. Recent systematic reviews and meta-analyses have strengthened the evidence that physical activity has beneficial effects on depressive and anxiety symptoms [9–11]. Havnen and colleagues [12] found that more physically fit persons had a significantly lower consumption of anxiolytics and antidepressants. Further, physical activity may have a positive effect on people suffering from psychotic disorders [13].

Previous studies have found that endurance exercise at a low intensity may reduce depressive symptoms and anxiety symptoms [14]. An example of low intensity endurance training is prolonged exercise at approximately 60% of maximal heart rate. A potential mechanism for the effects of performing such exercise is the secretion of neurochemical substances (endorphins, serotonin and dopamine), which may have a positive effect on mood [15].

A recent meta-analysis concluded that performing endurance exercise at a high intensity may also reduce anxiety and depression [16]. High intensity exercise is shorter in duration (often shorter than 30 minutes) and with a heart rate between 85%–95% of maximal heart rate [17]. High intensity exercise is often performed in interval form, consisting of intermittent bouts of high and low intensity, a method which has been used frequently in research in recent years.

A so-called “4 × 4” interval session consisting of four minutes of high intensity divided into four bouts is a popular way of performing high-intensity exercise [18]. Recent studies have however found that the difference between performing 1 × 4 (i.e. one bout of four minutes) and 4 × 4 is small and non-significant in terms of training effect, measured by maximal oxygen uptake [19].

However, there has been some uncertainty regarding whether high-intensity exercise is superior to low-intensity exercise for mental health benefits [20]. A previous study found high-intensity exercise to have a significantly better effect on anxiety, compared to exercising at a lower intensity [21].

A more recent review by Singh and colleagues [14] also found high intensity exercise to have a better effect on mental health than lower intensity exercise. Additionally, the anxiety-suppressant and anti-depressant effect of high intensity exercise may come about rapidly, often after days or weeks [14]. An assumed mechanism is a larger secretion of signalling substances (serotonin and norepinephrine) following high intensity exercise compared to low intensity exercise [22]. Thus, it is important to examine the effect of high intensity exercise on mental health and whether a 1 × 4-interval protocol may be recommended for patients in mental health care.

An advantage of high intensity exercise is the shorter time required to complete a session compared to low intensity exercise. A high intensity exercise session is often completed in 20–30 minutes, with two sessions per week being sufficient. Thus, high intensity exercise is a time-efficient way of exercising, shown to have a higher compliance than exercising at lower intensities [23].

The shorter duration and higher perceived effectiveness of this type of training may further support continued exercise behaviour after the intervention period. High-intensity exercise may also lead to a greater reduction in blood pressure compared to low-intensity exercise [5, 14, 20], caused by improved autonomic regulation and cardiovascular adaptation. Changes in blood pressure may represent an important physiological marker of response.

Although the beneficial effects of exercise on anxiety are well documented, the optimal exercise intensity for reduction of anxiety is still unclear. This study therefore aims to provide new evidence on how exercise intensity influences both psychological and physiological outcomes. Together, these aspects represent the novel contribution of the present trial.

The main objective of this randomised controlled trial (RCT) is to evaluate the effectiveness of a four-week intervention involving high-intensity exercise, compared to low-intensity exercise. We hypothesise that the primary outcome measures (the Beck Anxiety Inventory [24] and the Hospital Anxiety and Depression Scale [25]) will show improvement as a result of the exercise. Furthermore, we also hypothesise that high-intensity exercise will lead to a significantly greater reduction in symptoms compared to low-intensity exercise.

## Hypotheses/aims and benefits

The aim of the current RCT is to examine whether high-intensity exercise may reduce symptoms of anxiety in patients in mental health care.

We hypothesise that:

1. The participants performing high-intensity exercise will have a significantly larger reduction in anxiety levels compared to the participants performing low-intensity exercise.
2. The participants performing high intensity exercise will have a significantly higher degree of compliance (meaning that they will be able to complete the training autonomously three months after participating in the project) compared to the participants performing low intensity exercise.
3. The participants performing high-intensity exercise will have a significantly larger improvement (i.e. reduction) in blood pressure than the participants performing low-intensity exercise.

The expected benefits for the participants are positive health effects, in terms of improved physical fitness and reduced psychological symptoms. Moreover, the participants will learn to use physical activity as a specific measure that they can utilise as a source of coping. Furthermore, knowledge about physical activity and mental health may be applicable in mental health care as part of a treatment plan.

## Methods/design

This is a study protocol for a randomised controlled trial designed to test the effects of high intensity training compared to low intensity training on anxiety levels.

There will be a random 1:1 allocation into two groups. The two groups are high intensity exercise (1 × 4 minutes performed two times per week with a heart rate of 85–95% of maximal heart rate, corresponding to ≥ 16 on the 6–20 Borg scale) and low intensity exercise (45 minutes performed two times per week with a heart rate of approximately 60% of maximal heart rate, corresponding to 9–11 on the 6–20 Borg scale) [26]. The high intensity group will perform a 10-minute warm-up prior to the high-intensity bout, and a 5-minute cooldown. Due to the low intensity in the low intensity group, a warm-up is not considered necessary.

The total amount of training sessions for both groups will be eight. The high intensity exercise group will serve as the intervention group, while the low intensity exercise group will serve as an active control group.

The trial will be conducted in accordance with the Standard Protocol Items: Recommendations for Interventional Trials statement (SPIRIT) [27].

The trial has been approved by the Data Protection Officer at the University Hospital of North Norway and the Regional Committee on Health Research Ethics (approval number 714458) and registered at ClinicalTrials.gov (Trial registration number NCT06881758).

Participants will provide written informed consent before allocation to the two intervention groups, and they will be randomised into a high-intensity training group and a low-intensity training group, following baseline measures.

Randomisation will be performed using counterbalanced block randomisation in a 1:1 ratio, stratified by sex, to ensure an equal distribution of males and females across the study groups. Block sizes of four–six participants will be used. This will allow for a balanced representation of both genders in each condition. Randomisation will be conducted using a computer-generated random number sequence via the website https://www.randomizer.org/.

**Fig. 1:**
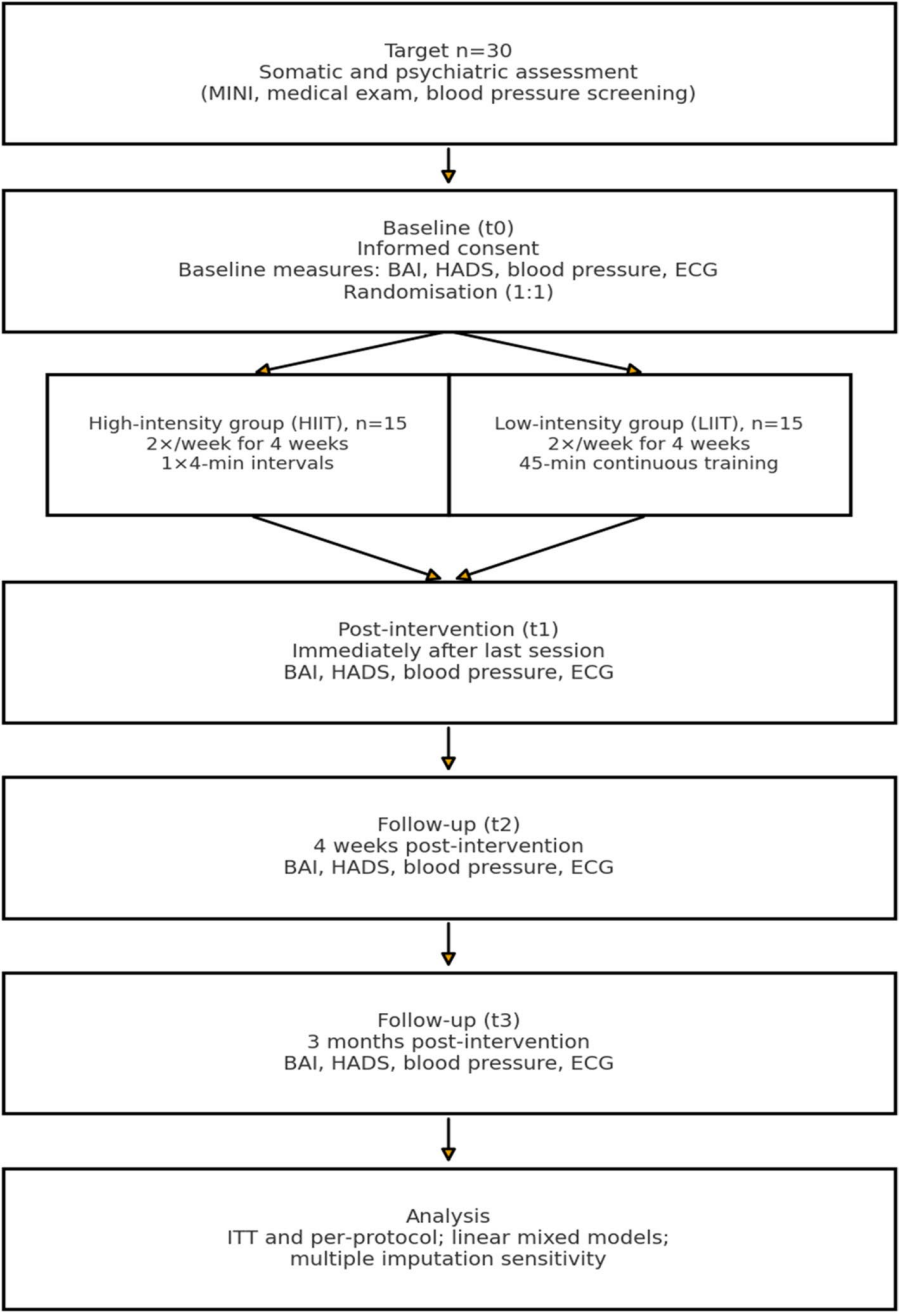
Study procedure flow chart

Outcomes will be measured immediately after inclusion (baseline), right after the very last exercise session, four weeks after the last exercise session (primary endpoint) and three months after the termination of the intervention period (secondary endpoint) (Fig. 1).

This approach allows for the assessment of the short-term effects of intervention and any residual effects.

Before the baseline measures, the participants are familiarised with the experimental procedures and devices used.

Table 1 is a SPIRIT diagram for trial stages of enrolment, intervention and assessment.

**Table 1:** SPIRIT diagram for trial stages of enrolment, intervention and assessment

	**STUDY PERIOD**				
	**Enrolment**		**Post-allocation**		
TIMEPOINT**	***Pre –enrolement***	**Baseline**	***t***_***1***_	***t***_***2***_	***t***_***3***_
**ENROLMENT:**					
**Eligibility screen**	X				
**Written informed consent**		X			
***Randomization***	X				
**ASSESSMENTS:**					
***Demograpichs***	X	X			
***MINI***		X			
***BAI***		X	X	X	X
***HADS***		X	X	X	X
***Blood pressure***		X	X	X	X
***EKG***		X	X	X	X

*MINI* Mini International Neuropsychiatric Interview [28], *BAI* Beck Anxiety Inventory [24], *HADS* Hospital Anxiety and Depression Scale [25], *EKG* electrocardiogram

t_1_ = right after the very last exercise session

t_2_ = four weeks after the last exercise session

t_3_ = three months after the termination of the intervention period

## Study setting

The study will take place at the Division of Substance Use and Mental Health at the University Hospital of North Norway, UNN-Åsgård, 9291 Tromsø, Norway.

The study participants will be examined by medical staff (doctors) to assess eligibility for participation in the study. The screening will include a clinical interview where the MINI-interview will be used [28]. The following descriptive characteristics will be collected: sex, age, body weight, height, waist circumference, blood pressure, pulse, respiratory frequency, temperature, somatic and psychiatric diagnoses and current medication.

Furthermore, a medical examination will be performed in order to ensure the patient can handle the physical training. In addition, a resting state EKG will be taken, a blood sample will be drawn, and the following analyses will be performed: haemoglobin, haematocrit, HbA1c, electrolytes, liver function tests, kidney function tests, TSH and fT4, lipids and PRO-BNP.

Following inclusion in the study, a study nurse will assist the participants in performing the psychometric tests.

At the study Hospital, there are two separate rooms for physical training, fitted with treadmills and other equipment for physical training. The interventions (physical training) will be carried out with the supervision of an exercise physiologist.

The treadmills are of the type Lexco T560 (Lexco, South Korea) and the patients will also be fitted with heart monitor belts of the type Polar H10 (Polar Electro, Finland).

## Study group

The project will be interdisciplinary where nurses, medical doctors, psychiatrists, psychologists and exercise physiologists will be cooperating.

The project is headed by Research Leader Martin Bystad, Ph.D., Clinical Psychologist at the Division of Mental Health and Substance Use at the University Hospital of North Norway and assistant professor at UiT The Arctic University of Norway.

The University Hospital of North Norway and UiT The Arctic University of Norway are represented through the participation, in an advisory role, of Psychiatrist/Professor Rolf Wynn, M.D., Ph.D.

Sveinung Rydland, M.Sc., Exercise Therapist at the Division of Mental Health and Substance Use, University Hospital of North Norway, will be responsible for conducting/supervising the training sessions.

Study Nurse Sissel Høgmo will function as coordinator and also administer psychometric instruments, take the EKG test, and perform physical measurements (as detailed below).

Dr. Christian Bugge, Dr. Birgit Brøndbo and Dr. Renate Jacobsen will perform the diagnostic assessments with MINI and the physical examinations of those being assessed for inclusion. The study doctors will determine whether participants can be included in the study based on data from the diagnostic assessment, history-taking and results from the somatic tests (including physical examination and physical measurements, EKG-test and blood tests). 

Cooperation with other national research environments will include St. Olav’s Hospital, where high intensity exercise is used as part of the treatment in mental health care. We will also cooperate with the National Network for High Intensive Training for Medical Purposes (organised by the Norwegian Directorate of Health).

International cooperation will involve CIBERSAM researchers from the University of Miguel Hernandez, Alicante, the European University of Madrid, and the Complutense University of Madrid, Spain.

## Trial coordination and oversight

The trial is coordinated by the Division of Mental Health and Substance Use at the University Hospital of North Norway, which also serves as the sponsor institution. The principal investigator (M.B) has overall responsibility for the scientific and ethical conduct of the study, including recruitment, data integrity and adherence to the approved protocol.

Coordination and follow-up of study activities are handled by the study coordinator (S.H), who oversees scheduling, data collection and communication among study staff. The principal investigator and study coordinator meet regularly to review study progress, recruitment and safety issues. Given the limited scope and low-risk nature of this single-site trial, no formal Trial Steering Committee or independent monitoring group has been established. Oversight is provided by the sponsor and the Regional Committee for Medical and Health Research Ethics (REK North) in line with applicable Norwegian regulations.

## Trial oversight and quality assurance

As this is a small-scale, single-site clinical study involving a low-risk behavioural intervention, no formal Data Monitoring Committee or external auditing procedures are established.

Oversight of the study is carried out by the authors who are responsible for monitoring recruitment progress, data quality and adherence to the approved protocol. They will hold regular internal meetings to review study progress and ensure that the study follows ethical and institutional guidelines.

Any protocol deviations or adverse events will be reported to the Regional Committee for Medical and Health Research Ethics (REK North).

## Participants

### Inclusion criteria

Eligible participants are patients aged 18–70 years, with anxiety disorders in accordance with ICD-10 criteria, including (but not limited to) generalised anxiety disorder, agoraphobia, panic disorder, social phobia, post-traumatic stress disorder, hypochondria and obsessive–compulsive disorder. Comorbidity will often occur, so the patient may also have symptoms of depression. However, the main disorder should be anxiety.

### Exclusion criteria

The exclusion criteria are somatic diseases that may impede training or where training may have a detrimental effect, for instance severe cardiovascular disease, severe asthma, severe COPD, cancer and poorly regulated diabetes. People with severe mental disorders such as schizophrenia or bipolar disease, and people with an increased risk of suicide, and those with substance addiction, will not be eligible for inclusion. People using anxiolytics (benzodiazepines) and sleeping medication (z-hypnotics, melatonin, antihistamines) can be included provided this medication is used infrequently and in small doses.

### Intervention group

The intervention comprises 4 weeks of high-intensity exercise. Two training sessions will be completed each week, with a total of 8 sessions during the intervention period. Each session will include running or walking (depending on the participant’s level of fitness) on the treadmill for 4 minutes at a high level of intensity. The intensity is measured by the participant’s heart rate, which should be above 85% of maximal heart rate, and a rating of perceived exertion using the Borg scale of ≥ 16. To ensure sufficient intensity, the treadmill will be set at a minimum of 5%, similar to previous studies on interval training [17].

Participants will otherwise be encouraged to maintain their daily routine but refrain from changing their current pharmacological treatment or initiating other physical exercise practices during the study.

The high intensity exercise group will serve as the intervention group, while the low intensity exercise group will serve as an active control group.

### Comparison group

The comparison group comprises 4 weeks of low-intensity exercise. Two training sessions will be completed each week, with a total of 8 sessions during the intervention period. Each session will include running or walking (depending on the participant’s level of fitness) on the treadmill for 45 minutes at a low level of intensity. The intensity is measured by the participant’s heart rate, which should be approximately 60% of maximal heart rate, and a rating of perceived exertion using the Borg scale of 9–11.

Participants will otherwise be encouraged to maintain their daily routine but refrain from changing their current pharmacological treatment or initiating other physical exercise practices during the study.

## Outcomes

### Primary outcome measures

The primary outcome measure will be the degree of anxiety symptoms measured by the Hospital Anxiety and Depression Scale (HADS) [25] and the Beck Anxiety Inventory (BAI) [24] prior to the first (pre) and after the last (post) training session is completed.

The HADS is a questionnaire that measures self-report levels of some symptoms of anxiety and depression [25]. The instrument consists of 14 questions (items), of which 7 are related to anxiety symptoms (HADS-A) and 7 to depression symptoms (HADS-D). Each item is rated from 0 to 3, which gives a range of scoring from 0 to 42, with a score ranging between 0 and 21 for the anxiety (HADS-A) and depression (HADS-D) subscales. The HADS is one of the most commonly used self-report instruments for measuring anxiety and depression [29] and has demonstrated good reliability and validity and has sound psychometric properties [29]. A previous study found a minimal clinically important difference (MCID) between 0.5 and 5.57 for the HADS-D, and between 0.81 and 5.21 for HADS-A [30]. We will calculate total HADS scores and subscores for anxiety symptoms and depressive symptoms, respectively.

The Beck Anxiety Inventory (BAI) [24] is a questionnaire that measures self-report levels of some symptoms of anxiety. The instrument consists of 21 questions (items), and each item is rated from 0 to 3, which gives a range of scoring from 0 to 63, a higher score indicating increased symptoms. The BAI measures anxiety symptoms including muscle tension, dizziness, racing heart, nervousness, fear of dying, abdominal discomfort, face flushing, sweating, light-headedness, fear of the worst happening and a feeling of choking. The BAI is one of the most commonly used self-report instruments for measuring anxiety [31] and has demonstrated good reliability and validity and has sound psychometric properties [32]. We will calculate total BAI scores.

Both the Hospital Anxiety and Depression Scale (HADS) and the Beck Anxiety Inventory (BAI) will be applied. HADS allows comparability with both psychiatric and somatic populations, while BAI provides a more detailed assessment of somatic anxiety symptoms. Using both HADS and BAI increases sensitivity and also validity in assessing anxiety.

### Secondary outcome measures

#### Compliance

Compliance, i.e. to what degree the participants are able to sustain the exercise regimen three months after participating in the project, will be one secondary outcome measure.

#### Change in blood pressure

Blood pressure will be measured as a secondary outcome, reflecting physiological stress response and cardiovascular health. As the link between blood pressure and anxiety remains underexplored, this study may provide novel insights into this relationship.

Participants’ changes in blood pressure, i.e. any difference between blood pressure measured at inclusion and at the end of the 4-week intervention, will be a secondary outcome measure.

Blood pressure at baseline is also part of the medical screening to exclude participants with very high blood pressure not compatible with exercise participation.

#### Heart rate variability

Heart rate variability (HRV), derived from ECG recordings, will be applied as a physiological indicator of autonomic regulation and stress response. HRV provides an objective complement to the self-reported anxiety measures (HADS and BAI), which measure the participants’ subjective experience of anxiety symptoms.

#### Adherence

Adherence will be assessed by the number of completed sessions and heart-rate data compliance. The training schedule is flexible, allowing participants to make up missed sessions in subsequent weeks to support adherence.

## Sample size calculation

The sample size calculation of the present study was informed by data from a previous study [21] on psychological effects of high-intensity exercise, which showed a significant reduction in anxiety levels in 33 participants with anxiety disorders. We conducted a power calculation using https://clincalc.com/stats/samplesize.aspx. We assumed a baseline mean of 30 on the Beck Anxiety Inventory (BAI) for each group. The expected post-treatment BAI scores are 14 for the high-intensity group and 22 for the low-intensity group. With a significance level (alpha) of 0.05, SD of 7 and 80% power, the required sample size is 24. This means we need 12 participants per group to detect significant differences using a two-tailed *t*-test. Previous studies have found that the average attrition rate is 20% [33].

To account for an expected attrition rate of about 20% [33], the final target sample size is 30 participants (15 per group), representing the minimum required for adequate power. Additional participants may be included if recruitment permits, to further strengthen statistical power. Potential missing data will be addressed using linear mixed-model analyses, since this analysis is robust to missing-at-random mechanisms. Sensitivity analyses based on both intention-to-treat and per-protocol datasets will be applied to assess the robustness of the findings.

### Recruitment

The participants will be recruited from two sources: from a waiting list at the Outpatient Clinic and by direct contact.

Patients aged 18–70 that are referred for anxiety disorders and who are currently on the waiting list for care at the Adult Psychiatric Outpatient Clinic at the Division of Substance Use and Mental Health will be one source of recruitment. The waiting list is long, and participation in the project will represent a great advantage for participants while waiting to receive treatment at the Hospital’s Outpatient Clinic. A secretary will distribute invitational letters. Participation in the project will have no impact on the participants’ waiting list status. Patients that have had their referral to the outpatient clinic rejected, but who fulfil the study’s inclusion criteria, will also be invited to participate.

Active recruitment via media is an additional strategy to recruit people suffering from anxiety. This recruitment strategy may reduce selection bias, in the sense that we will be able to include people who are not currently receiving treatment for anxiety by their general practitioners. The study has been presented in news stories online and in print media, and people with anxiety disorders (diagnosed or non-diagnosed) may contact the study organisers directly by phone or e-mail to discuss their participation.

Information about the study and screening for eligibility after verbal consent is carried out by phone. If the screening criteria are met and the person is interested in participating in the study, an appointment is scheduled. Potential participants will receive the participant information orally and in writing.

Prior to participation, a screening will be performed in order to examine whether potential participants fulfil the inclusion criteria and that the exclusion criteria are not met. The screening will include a clinical interview with a medical doctor, where the MINI-instrument will be used. A medical doctor will take the participant’s medical history, including but not limited to the elements of past history, natural functions and current medication.

Furthermore, a general medical examination including a clinical assessment of the cardiovascular, pulmonary, musculoskeletal and neurological systems will be performed by a medical doctor in order to ensure the patient can partake in the physical training safely. The study nurse will carry out the measurement of blood pressure, pulse, respiratory frequency, temperature, weight and height and waist circumference. A resting state EKG will be taken by the study nurse and assessed by a medical doctor. The following blood tests will be taken: haemoglobin and haematocrit, HbA1c, electrolytes, liver function tests, kidney function tests, TSH and fT4, lipids and PRO-BNP.

A medical doctor will, following the completion of examinations outlined above and the results of the tests, make an assessment regarding inclusion or exclusion in the study. Patients that are excluded for medical reasons will be followed up by the doctor and/or referred to another medical doctor for follow-up.

### Blinding

The study is not double-blinded, as this is not possible with the current intervention. However, the study nurse is blinded to the condition (high-intensity vs low-intensity), while the exercise physiologist is blinded to the scores on the outcome measures. Therefore, the study nurse does not know which condition the participants belong to, while the exercise physiologist has no insight into how the patients score on the various outcome measures.

### Participant adherence to the intervention, adverse events and concomitant care

The exercise physiologist will make appointments with the participants to perform the training interventions, and he will support adherence by following up on the participants, monitoring any adverse effects and providing real-time feedback.

Serious adverse events will be reported to the North Norway Regional Ethics Committee in accordance with the Health Research Act. Physical exercise is generally considered safe [34]; hence, no adverse events are expected; however, possible harms (e.g. injuries or severe events) are monitored using baseline values to oversee the condition of all participants during the trial.

Although the intervention is considered low risk, all participants will be continuously monitored for potential adverse events during each training session. The exercise physiologist and study nurse will record and report any untoward medical occurrences (e.g. cardiovascular symptoms, injuries, or other health-related complications).

In the event of any serious somatic or medical incident, the participant will immediately be withdrawn from the study and receive appropriate medical evaluation and treatment. The trial will be temporarily suspended or stopped if such events indicate that continued participation poses a health risk to any participant.

Serious adverse events will be reported to the Regional Committee for Medical and Health Research Ethics (REK North) and the Data Protection Officer in accordance with the Norwegian Health Research Act.

Given the short duration and low-risk nature of the intervention, no formal interim analyses or statistical stopping rules have been defined. The intervention is considered a low-risk and natural physical activity, similar to standard exercise routines recommended for the general population. Therefore, the likelihood of any serious adverse medical event is regarded as minimal.

### Statistical analysis

Data management and analyses are performed using SPSS 30 (SPSS Inc., Chicago, IL, USA). Data will be analysed using descriptive and inferential statistics. Continuous variables are presented with mean ± SD and 95% CIs.

The effect of the intervention will be assessed by examining change scores on the BAI and HADS (primary outcomes) and blood pressure and EKG (secondary outcomes). Changes from baseline to post-tests (immediately, after four weeks, and after three months) will be analysed.

Single measures (e.g. baseline data) will be analysed using ANOVA or linear regression, provided the statistical assumptions for linear models are met. Variables will be assessed for normality through visual inspection of histograms and Q-Q plots, along with a formal test for normality (Shapiro-Wilk test). If the assumption of normality is violated, non-parametric tests will be applied. ANOVA with repeated measures will be used to analyse changes in quantitative data (change scores) and to compare the two groups.

Missing data will be handled using linear mixed-model analyses, which provide unbiased estimates under the assumption that data are missing at random and allow inclusion of all available observations. Furthermore, multiple imputation will be applied as a sensitivity analysis to assess the robustness of the results to potential missingness.

We will also use Bonferroni corrected post hoc comparisons when performing multiple comparisons, ensuring that the overall significance level remains accurate. Additionally, effect sizes are estimated based on Cohen’s *d* (0.2 = small effect, 0.5 = medium effect, and 0.8 = large effect).

Participants with mild-to-moderate depressive symptoms will be eligible for inclusion, as anxiety and depression frequently co-occur in clinical populations. Due to our limited sample size, formal subgroup analyses based on depression status will not be conducted. Instead, the baseline depressive symptom scores (HADS-D) will be entered as a covariate in the statistical models to adjust for their potential influence on anxiety outcomes.

Age will be included as a covariate in all primary and secondary analyses. In addition, exploratory subgroup analyses will be performed by age group (< 40 years vs ≥ 40 years).

## Patient and public involvement

A user representative (Ms. Line Wold Gudvangen) participated in the planning phase of the project. She reviewed the study protocol and participant information materials, and provided advice on the feasibility and participant burden of the intervention. Although patients or members of the public were not directly involved in the design of the research question, user input has been important for clinical relevance and for planning future implementation of the findings.

## Discussion

Prior studies have found that physical exercise is beneficial for patients suffering from different types of psychiatric illnesses. Exercise may of course improve physical health but can also contribute to a reduction in psychiatric symptoms.

While prior studies have suggested that high-intensity training can lead to a reduction in anxiety, there is less evidence relating to the benefits of this type of intervention within the frame of mental health services.

Moreover, little is known about how high intensity training compares to low intensity training with respect to effects on anxiety levels in patients suffering primarily from anxiety disorders.

This is, to our knowledge, one of the first studies that compares these two different types of physical training for patients suffering from anxiety disorders.

Participants will be recruited by contacting those referred to the Study Hospital for outpatient treatment of anxiety and also by direct active recruitment through media.

Participants will undergo a thorough psychiatric and somatic examination to assess eligibility and to exclude those who may be physically unfit to participate. We will randomise the participants into a high-intensity training group and a low-intensity training group, in order to study whether the intensity of training is of importance to outcome in terms of reduced anxiety symptoms.

The duration (four-week intervention) was chosen based on feasibility within the clinical setting, but also previous evidence that high-intensity interval training (HIIT) can yield measurable psychological and physiological benefits within short timeframes [21]. This supports a short, structured and time-efficient design suitable for clinical practice.

Complete blinding is not feasible or possible in exercise interventions, as participants and trainers are aware of the training intensity. To minimise bias, however, the outcome assessor (study nurse) will be blinded to group allocation, and the exercise physiologist will not have access to any outcome data.

This study will add to the present-day understanding of the efficacy of physical training for people suffering from anxiety, and for the importance of physical training for mental health in general.

The knowledge generated by this study may directly impact the evidence towards developing targeted interventions and the self-management of anxiety disorders, and the knowledge will be of importance in developing recommendations for the treatment of patients suffering from anxiety disorders. Should the treatment prove effective, this will be a simple, convenient and low-cost treatment option for many people.

## Ethics and dissemination

The project complies with the relevant national regulations and institutional policies and will be performed following the Helsinki Declaration.

The trial is approved by the Data Protection Officer at the University Hospital of North Norway and the Regional Committee on Health Research Ethics (714458) and has been registered at ClinicalTrials.gov (NCT06881758). The protocol and the ClinicalTrials.gov registration together include all items specified in the WHO Trial Registration Data Set, except for trial results, which will be made available after study completion.

The participants will be informed of the study objectives, risks and benefits and must provide written informed consent before participation. Participants can stop participation at any time without giving an explanation.

The study findings will be disseminated through international and national peer-reviewed journals, at national and international conferences, and through media releases. The results will be submitted for publication irrespective of the study findings.

Any substantial changes (i.e. changes affecting participant safety, scientific validity, study procedures, or data protection) will be submitted to the Regional Committee for Medical and Health Research Ethics (REK North; approval no. 714458) for approval before implementation. Urgent safety measures may be implemented immediately and will be notified to REK without delay. Changes that affect data processing will be notified to the Data Protection Officer at the University Hospital of North Norway, and ClinicalTrials.gov (NCT06881758) will be updated.

## Data statement section

Data (including diagnostics, psychometrics, results from blood tests and training data) for each participant will be securely stored in the Electronic Health Records at the Study Hospital. Source documents, including dates and participant IDs, will be scanned and saved as electronic copies, and the paper material will be destroyed.

In the data-analysis process, each participant will be given a unique ID code and the corresponding ID will be kept separate in a secure location.

Data will be stored for five years after the termination of the trial after which the data will be anonymised.

## Trial status

The protocol version number and date: Version number 1.1, 01/11/2025.

Recruitment: Recruitment started in January 2025. The anticipated recruitment completion is in March 2026.

## Supplementary Information

Supplementary Material 1.

## Data Availability

The full trial protocol and informed consent materials are available at any reasonable request to the corresponding author. The final trial dataset and the code for statistical analysis will be accessible by non-commercial partners upon reasonable request.

## References

[1] Wilmer MT, Anderson K, Reynolds M. Correlates of quality of life in anxiety disorders: review of recent research. Curr Psychiatry Rep. 2021;23(11):77. 34613508 10.1007/s11920-021-01290-4 PMC8493947

[2] Reneflot A, aarø LE, Aase H, Reichborn-Kjennerud T, Tambs K, Øverland SN. Psykisk helse i Norge. 2018.

[3] Bandelow B, Michaelis S. Epidemiology of anxiety disorders in the 21st century. Dialogues Clin Neurosci. 2015;17(3):327–35. 26487813 10.31887/DCNS.2015.17.3/bbandelow PMC4610617

[4] Smith PJ, Merwin RM. The role of exercise in management of mental health disorders: an integrative review. Annu Rev Med. 2021;27(72):45–62. 10.1146/annurev-med-060619-022943 PMC8020774 33256493

[5] Piercy KL, Troiano RP, Ballard RM, Carlson SA, Fulton JE, Galuska DA, et al. The physical activity guidelines for Americans. JAMA. 2018;320(19):2020–8. 30418471 10.1001/jama.2018.14854 PMC9582631

[6] Bahr R. Aktivitetshåndboken. Fysisk aktivitet i forebygging og behandling. 2009.

[7] Helsedirektoratet. Anbefalinger om kosthold, ernæring og fysisk aktivitet. Helsedirektoratet; 2022.

[8] Bull FC, Al-Ansari SS, Biddle S, Borodulin K, Buman MP, Cardon G, et al. World Health Organization 2020 guidelines on physical activity and sedentary behaviour. Br J Sports Med. 2020;54(24):1451–62. 33239350 10.1136/bjsports-2020-102955 PMC7719906

[9] Noetel M, Sanders T, Gallardo-Gómez D, et al. Effect of exercise for depression: systematic review and network meta-analysis of randomised controlled trials. BMJ. 2024;384:e075847. 10.1136/bmj-2023-075847. 38355154 10.1136/bmj-2023-075847 PMC10870815

[10] Lin Y, Gao W. The effects of physical exercise on anxiety symptoms of college students: a meta-analysis. Front Psychol. 2023;14:1136900. 10.3389/fpsyg.2023.1136900. 37063553 10.3389/fpsyg.2023.1136900 PMC10100500

[11] Jemni M, Zaman R, Carrick FR, Clarke ND, Marina M, Bottoms L, Matharoo JS, Ramsbottom R, Hoffman N, Groves SJ, Gu Y, Konukman F. Exercise improves depression through positive modulation of brain-derived neurotrophic factor (BDNF). A review based on 100 manuscripts over 20 years. Front Physiol. 2023;14:1102526. 10.3389/fphys.2023.1102526 PMC10030936 36969600

[12] Havnen A, Zotcheva E, Bjerkeset O, Sui X, Ernstsen L. Cardiorespiratory fitness and incident use of anxiolytics and antidepressants in adults. A linkage study between HUNT and the Norwegian Prescription Database. J Affect Disord. 2023;339:111–117. 10.1016/j.jad.2023.07.029 37437717

[13] Damme KSF, Sloan RP, Bartels MN, Ozsan A, Ospina LH, Kimhy D, et al. Psychosis risk individuals show poor fitness and discrepancies with objective and subjective measures. Sci Rep. 2021;11(1):9851. 33972634 10.1038/s41598-021-89301-5 PMC8110757

[14] Singh B, Olds T, Curtis R, Dumuid D, Virgara R, Watson A, et al. Effectiveness of physical activity interventions for improving depression, anxiety and distress: an overview of systematic reviews. Br J Sports Med. 2023;57(18):1203–9. 36796860 10.1136/bjsports-2022-106195 PMC10579187

[15] Erickson KI, Miller DL, Roecklein KA. The aging hippocampus: interactions between exercise, depression, and BDNF. Neuroscientist. 2012;18(1):82–97. 10.1177/1073858410397054. 21531985 10.1177/1073858410397054 PMC3575139

[16] Martland R, Korman N, Firth J, Vancampfort D, Thompson T, Stubbs B. Can high-intensity interval training improve mental health outcomes in the general population and those with physical illnesses? A systematic review and meta-analysis. Br J Sports Med. 2022;56(5):279–91. 34531186 10.1136/bjsports-2021-103984

[17] Helgerud J, Høydal K, Wang E, Karlsen T, Berg P, Bjerkaas M, et al. Aerobic high-intensity intervals improve VO2max more than moderate training. Med Sci Sports Exerc. 2007;39(4):665–71. 17414804 10.1249/mss.0b013e3180304570

[18] Weston KS, Wisløff U, Coombes JS. High-intensity interval training in patients with lifestyle-induced cardiometabolic disease: a systematic review and meta-analysis. Br J Sports Med. 2014;48(16):1227–34. 24144531 10.1136/bjsports-2013-092576

[19] Tjønna AE, Leinan IM, Bartnes AT, Jenssen BM, Gibala MJ, Winett RA, et al. Low- and high-volume of intensive endurance training significantly improves maximal oxygen uptake after 10-weeks of training in healthy men. PLoS ONE. 2013;8(5):e65382. 23734250 10.1371/journal.pone.0065382 PMC3667025

[20] Fuentes-García JP, Collado-Mateo D, Villafaina S. Effects of high-intensity interval training and moderate-intensity training on stress, depression, anxiety, and resilience in healthy adults during Coronavirus disease 2019 confinement: a randomized controlled trial. Front Psychol. 2021;24(12):643069. 10.3389/fpsyg.2021.643069 PMC7943442 33716913

[21] Plag J, Schmidt-Hellinger P, Klippstein T, Mumm JLM, Wolfarth B, Petzold MB, et al. Working out the worries: a randomized controlled trial of high intensity interval training in generalized anxiety disorder. J Anxiety Disord. 2020;76:102311. 33007710 10.1016/j.janxdis.2020.102311

[22] Gujral S, Aizenstein H, Reynolds CF 3rd, Butters MA, Erickson KI. Exercise effects on depression: possible neural mechanisms. Gen Hosp Psychiatry. 2017;49:2–10. 29122145 10.1016/j.genhosppsych.2017.04.012 PMC6437683

[23] Li F, Kong Z, Zhu X, Chow BC, Zhang D, Liang W, et al. High-intensity interval training elicits more enjoyment and positive affective valence than moderate-intensity training over a 12-week intervention in overweight young women. J Exerc Sci Fit. 2022;20(3):249–55. 10.1016/j.jesf.2022.05.001. 35646131 10.1016/j.jesf.2022.05.001 PMC9120050

[24] Beck AT, Epstein N, Brown G, Steer RA. An inventory for measuring clinical anxiety: psychometric properties. J Consult Clin Psychol. 1988;56(6):893–7. 3204199 10.1037//0022-006x.56.6.893

[25] Zigmond AS, Snaith RP. The hospital anxiety and depression scale. Acta Psychiatr Scand. 1983;67(6):361–70. 6880820 10.1111/j.1600-0447.1983.tb09716.x

[26] Borg GA. Psychophysical bases of perceived exertion. Med Sci Sports Exerc. 1982;14(5):377–81. 7154893

[27] Chan AW, Tetzlaff JM, Gøtzsche PC, Altman DG, Mann H, Berlin JA, et al. SPIRIT 2013 explanation and elaboration: guidance for protocols of clinical trials. BMJ. 2013;8(346):e7586. 10.1136/bmj.e7586 PMC3541470 23303884

[28] Sheehan DV, Lecrubier Y, Sheehan KH, Amorim P, Janavs J, Weiller E, Hergueta T, Baker R, Dunbar GC. The Mini-International Neuropsychiatric Interview (M.I.N.I.): the development and validation of a structured diagnostic psychiatric interview for DSM-IV and ICD-10. J Clin Psychiatry. 1998;59 Suppl 20:22–33. 9881538

[29] Leiknes K, Dalsbø T, Siqveland J. Psychometric assessment of the Norwegian version of the Hospital Anxiety and Depression Scale (HADS). Norwegian Institute of Public Health. 2016. 29320005

[30] Lemay KR, Tulloch HE, Pipe AL, Reed JL. Establishing the minimal clinically important difference for the Hospital Anxiety and Depression Scale in patients with cardiovascular disease. J Cardiopulm Rehabil Prev. 2019;39(6):E6–11. 30489438 10.1097/HCR.0000000000000379

[31] Rector NA, Arnold PD. Assessment of patients with anxiety disorders. In: Goldbloom DS, editor. Psychiatric clinical skills. Philadelphia: Mosby; 2006. p. 71–89.

[32] Muntingh AD, van der Feltz-Cornelis CM, van Marwijk HW, Spinhoven P, Penninx BW, van Balkom AJ. Is the Beck Anxiety Inventory a good tool to assess the severity of anxiety? A primary care study in the Netherlands Study of Depression and Anxiety (NESDA). BMC Fam Pract. 2011;4(12):66. 10.1186/1471-2296-12-66 PMC3224107 21726443

[33] Collins KA, Huffman KM, Wolever RQ, Smith PJ, Siegler IC, Ross LM, Hauser ER, Jiang R, Jakicic JM, Costa PT, Kraus WE. Determinants of dropout from and variation in adherence to an exercise intervention: the STRRIDE randomized trials. Transl J Am Coll Sports Med. 2022;7(1):e000190. 10.1249/tjx.0000000000000190 PMC9165469 35669034

[34] Posadzki P, Pieper D, Bajpai R, Makaruk H, Könsgen N, Neuhaus AL, et al. Exercise/physical activity and health outcomes: an overview of Cochrane systematic reviews. BMC Public Health. 2020;20(1):1724. 33198717 10.1186/s12889-020-09855-3 PMC7670795

